# Current trends and latest developments in echocardiographic assessment of right ventricular function: load dependency perspective

**DOI:** 10.3389/fcvm.2024.1365798

**Published:** 2024-07-01

**Authors:** Hideaki Nonaka, Indrek Rätsep, Nchafatso G. Obonyo, Jacky Y. Suen, John F. Fraser, Jonathan Chan

**Affiliations:** ^1^ Critical Care Research Group, The Prince Charles Hospital, Brisbane, QLD, Australia; ^2^ Faculty of Medicine, University of Queensland, Brisbane, QLD, Australia; ^3^ Department of Intensive Care, North Estonia Medical Centre, Tallinn, Estonia; ^4^ Wellcome Trust Centre for Global Health Research, Imperial College London, London, United Kingdom; ^5^ Clinical Research and Training Department, Initiative to Develop African Research Leaders/KEMRI-Wellcome Trust Research Programme, Kilifi, Kenya; ^6^ Intensive Care Unit, St Andrews War Memorial Hospital, Brisbane, QLD, Australia; ^7^ Department of Cardiology, The Prince Charles Hospital, Brisbane, QLD, Australia; ^8^ School of Medicine and Menzies Health Institute Queensland, Griffith University, Gold Coast, QLD, Australia; ^9^ Faculty of Health Science and Medicine, Bond University, Gold Coast, QLD, Australia

**Keywords:** echocardiography, right ventricular (RV), right ventricular (RV) failure, strain, speckle tracking echocardiograph, myocardial work, right ventricular pulmonary artery coupling

## Abstract

Right ventricle (RV) failure is a common complication of many cardiopulmonary diseases. Since it has a significant adverse impact on prognosis, precise determination of RV function is crucial to guide clinical management. However, accurate assessment of RV function remains challenging owing to the difficulties in acquiring its intricate pathophysiology and imaging its complex anatomical structure. In addition, there is historical attention focused exclusively on the left ventricle assessment, which has led to overshadowing and delayed development of RV evaluation. Echocardiography is the first-line and non-invasive bedside clinical tool for assessing RV function. Tricuspid annular plane systolic excursion (TAPSE), RV systolic tissue Doppler velocity of the tricuspid annulus (RV S'), and RV fractional area change (RV FAC) are conventional standard indices routinely used for RV function assessment, but accuracy has been subject to several limitations, such as load-dependency, angle-dependency, and localized regional assessment. Particularly, load dependency is a vexing issue, as the failing RV is always in a complex loading condition, which alters the values of echocardiographic parameters and confuses clinicians. Recently, novel echocardiographic methods for improved RV assessment have been developed. Specifically, “strain”, “RV-pulmonary arterial (PA) coupling”, and “RV myocardial work” are newly applied methods for RV function assessment, a few of which are designed to surmount the load dependency by taking into account the afterload on RV. In this narrative review, we summarize the latest data on these novel RV echocardiographic parameters and highlight their strengths and limitations. Since load independency is one of the primary advantages of these, we particularly emphasize this aspect.

## 1 Introduction

Right ventricular (RV) failure is closely associated with poor prognosis and frequently coexists with various diseases, such as pulmonary embolism (PE), pulmonary artery hypertension (PAH), lung diseases such as Coronavirus disease 2019 (COVID-19), and left-sided heart failure (1, 2). To detect RV dysfunction early, guide appropriate therapy, and avoid poor clinical course, the precise assessment of RV function is essential (3, 4). Echocardiography is a versatile, non-invasive bedside imaging modality for evaluating RV function. According to the European Association of Cardiovascular Imaging (EACVI) reports, over 99% of clinicians routinely use echocardiography for the first-line assessment of the RV (5). Conventional parameters such as tricuspid annular plane systolic excursion (TAPSE), systolic tissue Doppler velocity of the tricuspid annulus (RV S’), and RV fractional area change (FAC) have been currently used for RV function evaluation; however, these conventional parameters have several limitations, one of which is the load-dependency. Their values change depending on the hemodynamic load exerted on the RV, leading to inaccurate assessment of RV function (6–8). Since a diseased RV is consistently under complex loading conditions, load dependency is a significant issue that must be addressed.

Traditionally, load-independent indicators of RV function have been obtained through invasive catheterization. End-systolic elastance (Ees), Ees/arterial elastance (Ea), and stroke work (SW) are gold standard parameters for accurate assessment of heart function in both clinical and research settings (9). However, the complexity of the analysis and the invasive nature of these measurements limit their widespread use.

**Figure 1:**
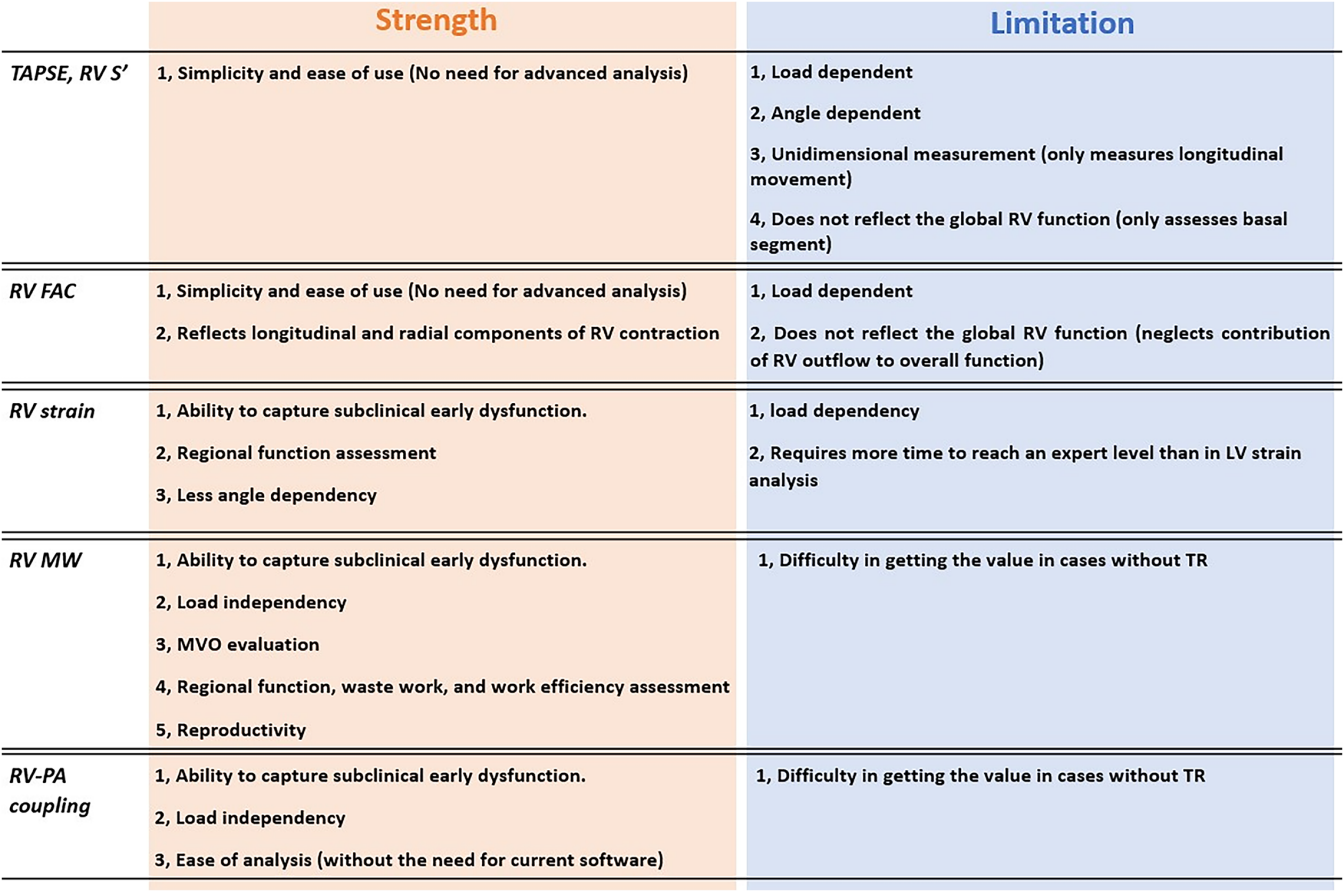
Strengths and limitations of novel and conventional RV echocardiographic parameters. RV, right ventricular; PA, pulmonary artery; RVD, right ventricular dysfunction; MVO, myocardial oxygen consumption; TR, tricuspid regurgitation.

Recently, new echocardiographic indicators of RV function have emerged, with the incorporation of loading conditions, allowing for a more accurate assessment of intrinsic RV contractility even in complex disease states. However, publications summarizing their characteristics and clinical evidence remain scarce. In this review, we have summarized the latest contemporary literature with a focus on load dependency and clinical utility. The strengths and limitations for each are outlined in Figure 1 and the load dependency has been reviewed in Table 1, mainly based on studies, in which load dependency was evaluated through correlations with invasive load-independent parameters such as Ees, Ees/Ea, and SW.

**Table 1:** Correlation with invasive parameters.

Echocardiographic parameter	Invasive parameter	Population	*N*	Study
Conventional parameters				
TAPSE	Ees (*r* = −0.28, *P* = 0.09) SW (*r* = −0.04, *P* = 0.82)	Post Fontan operation	42	Jana Schlangen et al. Circ CI, 2014 (6)
	Ees (*r* = 0.34, *P* = 0.176) Ees/Ea (*r* = 0.2, *P* = 0.07)	Dogs paced at 120–180 bpm	8	
RV FAC	Ees (*r* = 0.03, *P* = 0.85) SW (*r* = −0.07, *P* = 0.65)	Post Fontan operation	42	Jana Schlangen et al. Circ CI, 2014 (6)
RV strain				
RV GLS	Ees (*r* = 0.07, *P* = 0.5) SW (*r* = −0.13, *P* = 0.24)	Post Fontan operation	42	Jana Schlangen et al. Circ CI, 2014 (6)
	RV SWI (*R* = −0.27, *P* = 0.058)	PH (Group1,4)	51	Steele C. Butcher et al. Am J Cardiol, 2022 (10)
RV MW				
RV GCW	RV SWI (*R* = 0.63, *P* < 0.001)	PH (Group1,4)	51	Steele C. Butcher et al. Am J Cardiol, 2022 (10)
	RV SV (*r* = 0.63, *P* = 0.002) RV SVI (*r* = 0.59, *P* = 0.004)	HFrEF	44	Steele C. Butcher et al. EHJ CI, 2021 (11)
RV GWI	RV SWI (*R* = 0.60, *P* < 0.001)	PH (Group1,4)	51	Steele C. Butcher et al. EHJ CI, 2021 (11)
RV-PA coupling				
TAPSE/PASP	Ees/Ea (*r* = 0.71, *P* < 0.001)	HFrEF	110	Alexander Schemeisser et al. EHJ CI, 2021 (12)
	Ees/Ea (*r* = 0.044, *P* = 0.002)	PAH or CTEPH	52	Khodr Tello et al. Circ CI, 2019 (13)
	Ees/Ea (*r* = 0.498, *P* = 0.001)	PAH or CTEPH	38	Manuel J. Richeter et al. Am J Respir Crit Care Med, 2020 (14)
SV/ESA	Ees/Ea (*r* = 0.516, *P* < 0.001)	PAH or CTEPH	52	Khodr Tello et al. Circ CI, 2019 (13)
	Ees/Ea (*r* = 0.682, *P* < 0.001)	PAH or CTEPH	38	Manuel J. Richeter et al. Am J Respir Crit Care Med, 2020 (14)

In this article, we evaluated the load dependency of each parameter based on the correlation with invasive load-independent parameters such as Ees, Ees/Ea, and SWI.

TAPSE, tricuspid annular plane systolic excursion; RV FAC, RV fractional area change; RV GLS, right ventricular global longitudinal strain; PH, pulmonary hypertension; HFrEF, heart failure with reduced ejection fraction; PAH, pulmonary artery hypertension; CTEPH, chronic thromboembolic pulmonary hypertension; GCW, global constructive work; GWI, global work index; TAPSE, tricuspid annular plane systolic excursion; PASP, pulmonary artery systolic pressure; SV, stroke volume; ESA, end-systolic area; SWI, stroke work index; SV, stroke volume; SVI, stroke volume index; Ees, end-systolic elastance; Ea, arterial elastance.

In consultation with a medical librarian, we conducted a literature search in Medline Complete (EBSCOHost) and Embase (Elsevier) based on the concepts of novel echocardiographic parameters and right ventricular function (refer to Supplementary Table 1 for full search strategies). The publication date was limited from 1st January 2018 to 30th April 2023 because the major Joint American Society of Echocardiography (ASE)/EACVI Task Force recommendations on RV strain were published in January 2018 (15). In this review, we included studies that investigated three novel echocardiographic parameters: (1) RV strain, (2) RV myocardial work (MW), and (3) RV-PA coupling [TAPSE/pulmonary artery systolic pressure (PASP), RV S'/PASP, RVFAC/PASP, and RV longitudinal strain (LS)/PASP]. The database identified 2,442 papers, after excluding duplicates. Reviewers (HN, IR) excluded inappropriate literature sequentially and finally used 74 papers as references in this review (Flow diagram: Supplementary Figure 1). Additionally, we used 14 representative papers published before 2018 to comprehensively understand the physiology of the RV and the underlying principles of novel parameters.

## 2 Manuscript

### 2.1 Conventional echocardiographic parameters (TAPSE, RV S', RV FAC)

TAPSE, RV S', and RV FAC are common conventional indices of RV contractility. These parameters can be easily measured from an apical four-chamber view with conventional measurement methods such as M-mode or Tissue Doppler, and are widely used in clinical practice. However, these parameters have disadvantages such as load dependency, localized regional assessment, and angle dependency. Herein, we list some studies that provide evidence for their limitations.

Firstly, they are dependent on loading conditions. Changes in the preload or afterload can affect their values, making it difficult to capture the true intrinsic contractility of the RV. Yuichi et al. developed a healthy dog model and manipulated the preload by adjusting heart rate or respiratory rate. In this model, conventional parameters including TAPSE and RV FAC significantly decreased when the preload was reduced (7, 16). These studies demonstrated that conventional parameters are susceptible to changes in loading conditions. Hagdorn et al. reported that TAPSE remained unchanged in their chronic over-preloading rat model study, while Ees gradually decreased, revealing that TAPSE might miss RV dysfunction in chronic volume overloading conditions (8). Furthermore, we previously reported that TAPSE was not a significant predictor of mortality in a PH cohort because TAPSE can be pseudo-normalized by a lowered RV afterload in severe tricuspid regurgitation (TR) (17). In clinical settings, a diseased RV is invariably subjected to multiple loads, making load dependency a critical issue that requires careful consideration.

**Figure 2:**
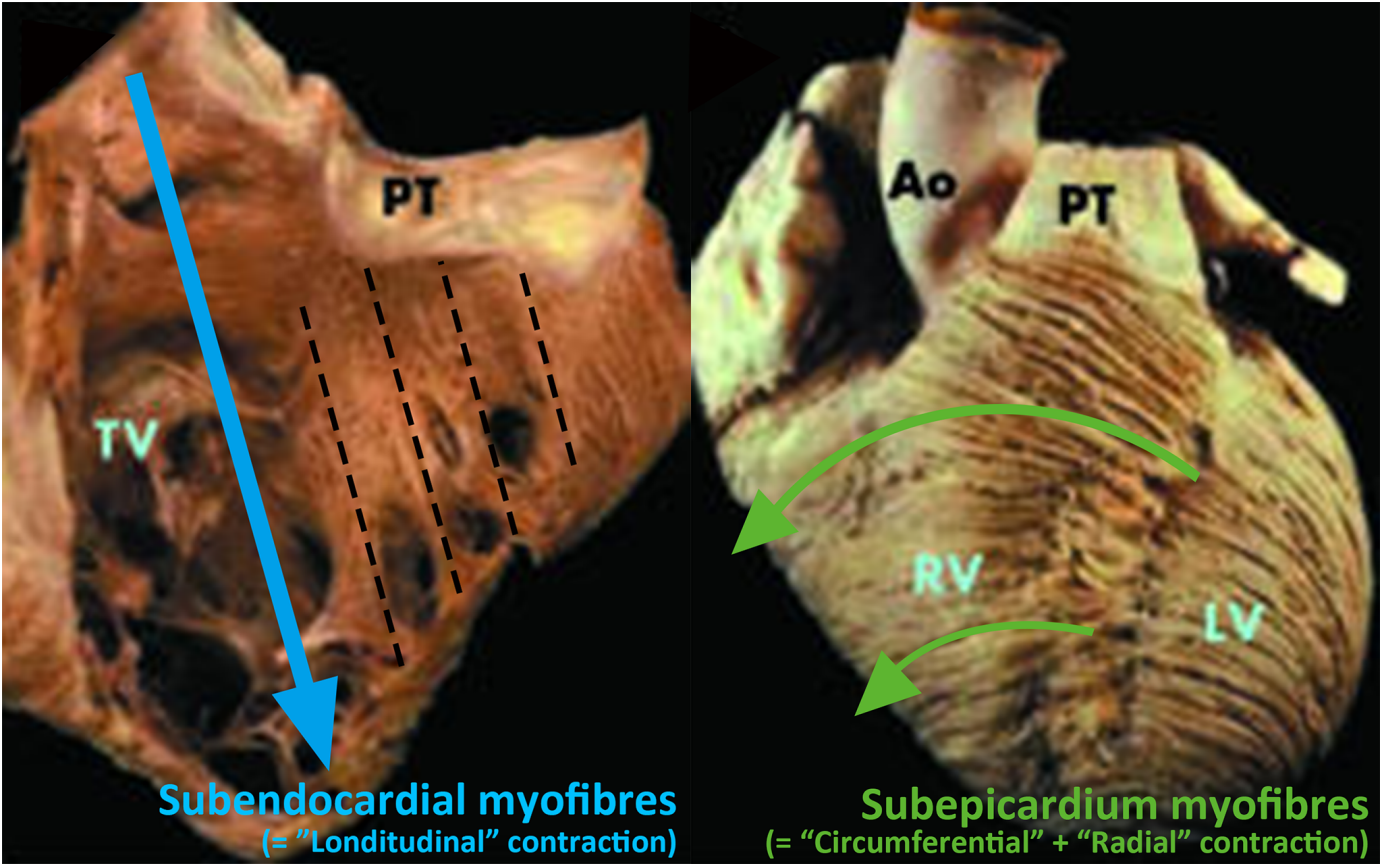
Orientation of RV myocardium fibers (18). Right ventricular has two myocardial layers. The first layer, subendocardial myofibers, is oriented longitudinally (left-hand panel) and the second layer, subepicardial myofibers, is oriented transversely from the superior to the inferior wall (right-hand panel). Reprinted with permission from Heart (18). ©2006 by BMJ Publishing Group Ltd via Copyright Clearance Center.

**Figure 3:**
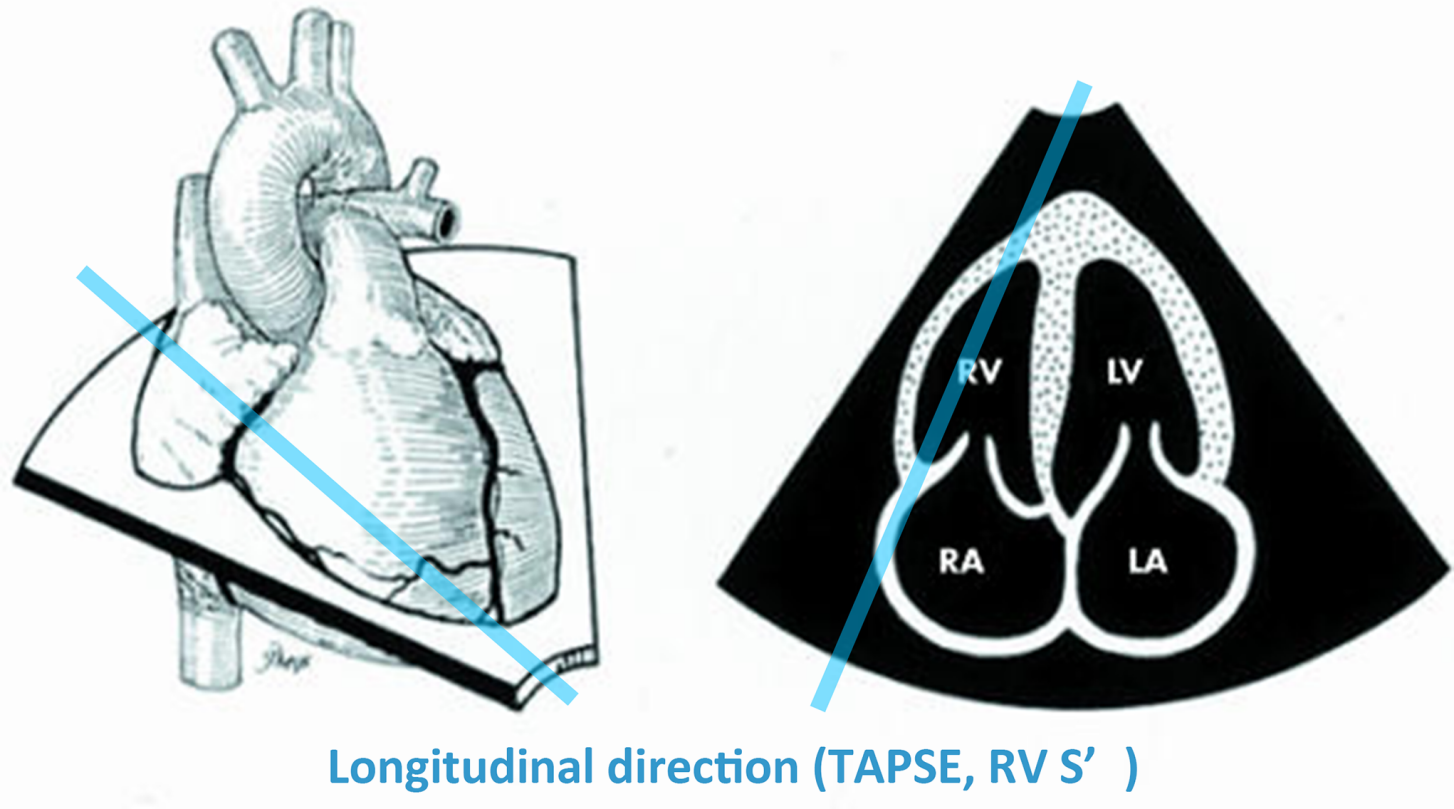
Evaluation of TAPSE and RV S’ limited by Doppler alignment (18). TAPSE, RV S’ can only assess longitudinal movement. Reprinted with permission from Heart (18). ©2006 by BMJ Publishing Group Ltd via Copyright Clearance Center.

Secondly, TAPSE and RV S' can only assess motion in a limited direction. RV is composed of two layers of myocardial fibers with longitudinal and transverse orientations that generate longitudinal, circumferential, and radial motion (18) (Figure 2). However, TAPSE and RV S' are primarily focused on measuring longitudinal motion (Figure 3).

Furthermore, TAPSE and RV S' can only assess RV function localized to RV basal segment and will not be able to identify regional RV dysfunction in pathologies such as arrhythmogenic RV cardiomyopathy (ARVC) and other forms of PH (17). In severe acute PE, the RV apex can be dysfunctional which can manifest as the Reverse McConnell's sign but this can be missed using TAPSE or RV S' alone (19).

**Figure 4:**
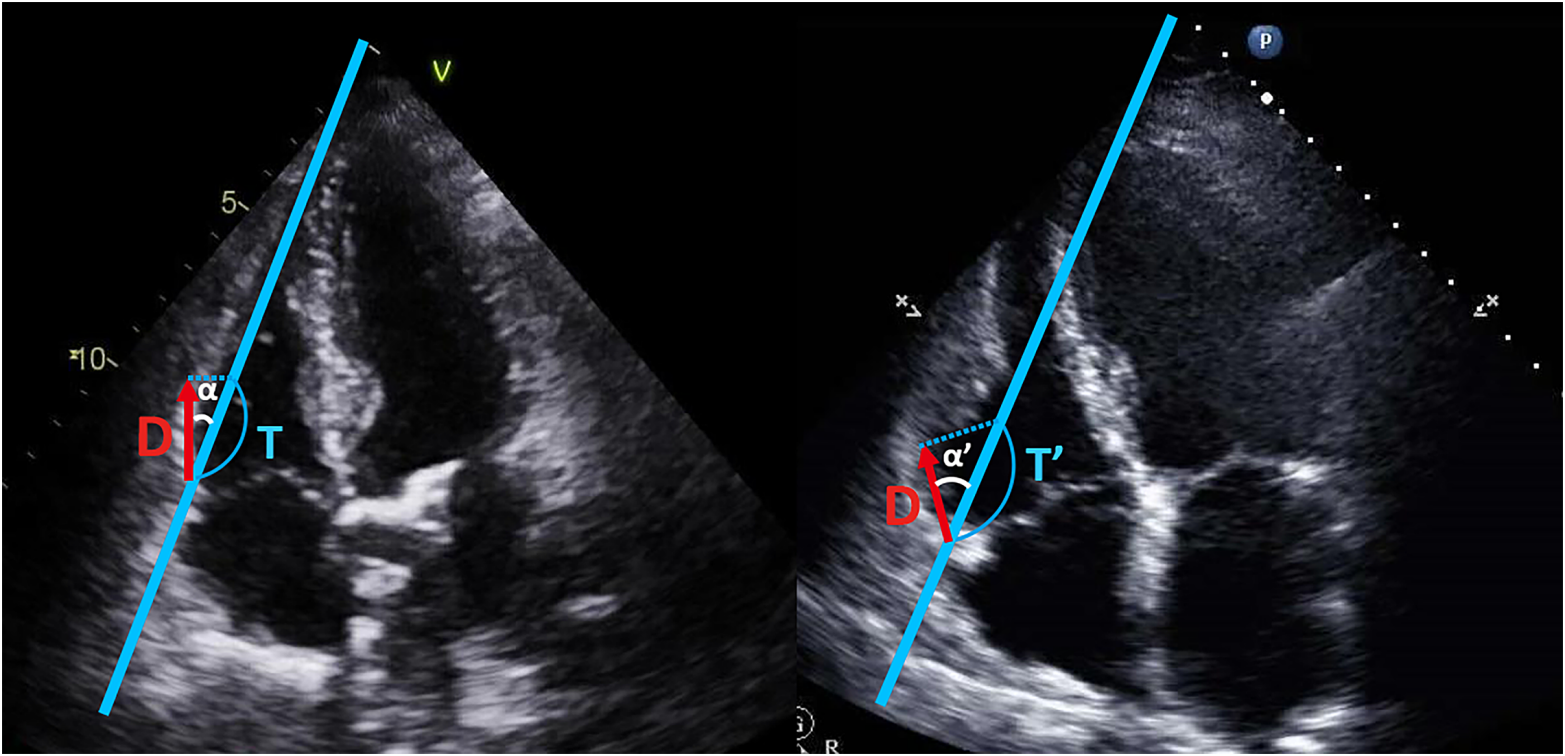
Angle dependency of TAPSE and RV S’. TAPSE and RV S’ are angle-dependent indices. The measured values (T, T’) for TAPSE and RV S’, depend on the cosine of angle α and α’. As the angle α increases to the angle α’, the value can be overestimated, as shown by the formulas below:$\begin{array}{rl} & T\;=\;D/\mathrm{cosine}\;\alpha ,\;T^{\prime }\;=\;D/\mathrm{cosine}\;\alpha^{\prime },\;90^{\circ }>\alpha^{\prime }>\alpha >0\\ & T^{\prime }>T \end{array}$. TAPSE, tricuspid annular plane systolic excursion; RV S’, systolic tissue Doppler velocity of the tricuspid annulus; D, the actual distance of longitudinal tricuspid annulus movement; T, T’, measured value (TAPSE, RV S’); α, α’, the angle between the ultrasound beam and the longitudinal axis of tricuspid annulus motion.

Regarding TAPSE and RV S', they have one additional significant limitation, angle dependency. Since two of them are Doppler techniques, these can be underestimated depending on the cosine of the angle between the ultrasound beam and the true tricuspid annulus motion, as shown by the formulas in Figure 4 (20).

Compared to TAPSE and RV S', RV FAC can evaluate both longitudinal and radial components of RV contraction. However, due to the RV complex crescent shape, even RV FAC may only assess partial movement andlike other 2-dimensional parameters including RV strain, fail to capture the contribution of the RV outflow tract to systolic function (21).

The reproducibility of each conventional parameter varies from the reports and methods to measure. In general, the reproducibility of TAPSE and RV S' is better than that of RV FAC due to its simplicity to measurements, yet novel parameters show better reproducibility primarily because of their automatic measurement nature by software (11).

In summary, conventional parameters are simple to evaluate; thus, they are widely used in clinical practice. However, their values can alter depending on the load conditions and the angle between the ultrasound beam and the heart's orientation. Additionally, they might not detect regional RV dysfunction, except at the base. These drawbacks are significant because they can cause confusion among physicians, potentially leading to misdiagnoses and inappropriate treatments. We will now proceed to discuss the novel RV parameters that overcome these limitations.

### 2.2 RV strain

Strain is the quantification of myocardial deformation, which can be measured using various imaging modalities. In echocardiography, there are two different measurement methods: the older “Tissue Doppler” derived strain and more contemporary “2D speckle-tracking echocardiography (STE)”. STE tracks the movement of “speckles”, caused by constructive interference from backscattered ultrasound waves, frame by frame in a 2-dimensional plane (22). Currently, RV strain measured by STE is increasingly being used as a novel echocardiographic parameter. Strain values are computed as the maximum myocardial deformation in systole. This enables the evaluation of cardiac function in three axes: longitudinal, circumferential, and radial, according to the “speckles” movement. Among these axes, the longitudinal (LS) is the most commonly used, and multiple studies have demonstrated the clinical utility of measuring RV LS in various pathologies including pulmonary artery hypertension (PAH), TR, congenital heart disease, cardiomyopathy including ARVC, and COVID-19 infection (Table 2).

**Table 2:** Predictive value and cut-off value of RV strain by STE.

Population	Study	*n*	Cut-off	Outcome	Analysis result	Comparison
PAH	Yuman Li et al. JASE, 2020 (23)	54		PAH-related hospitalization and death (median follow-up time was 28 months)	HR 1.19; 95% CI 1.03–1.45; *P* = 0.01	RV FAC, RV S’
PAH	Leah Wright et al. JACC CI, 2019 (24)	96		All-cause mortality (median follow-up time 13 months)	HR 0.90; 95% CI 0.83–0.97; *P* = 0.007	RV FAC, TAPSE
TR	Tom Kai Ming Wang et al. Circ CI, 2021 (25)	262		All-cause mortality (mean follow-up of 2.5 years)	HR 1.10; 95% CI 1.04–1.17; *P* = 0.001	
TR	Minkwan Kim et al. J Am Heart Assoc, 2021 (26)	115	**FWLS −24.0%**	Composite of cardiac death and unplanned readmission due to CV causes (5-years after surgery)	HR 2.30; 95% CI 1.22–4.36; *P* = 0.011	
TR	Marwin Bannehr et al. Can J Cardiol. 2021 (27)	1,089	**FWLS −18.0%**	All-cause 2-year mortality	HR 1.130; 95% CI 1.10–1.16; *P* < 0.001	RV FAC, TAPSE
TR	Francesco Ancona et al. EHJ CI, 2021 (28)	250	**FWLS −17.0%**	The presence of RVHF	AUC 0.66; sens 63%, spec 54%; *P* = 0.002	
			**FWLS −14.0%**	All-cause mortality (30 month follow-up)	AUC 0.70; sens 72%, spec 54%; *P* = 0.001	
TR	Rocio Hinoja et al. JASE, 2023 (29)		**FWLS −21.5%**	All-cause mortality and HFH (median follow-up period of 26 months)	AUC 0.82; sens 80%, spec 74%; *P* < 0.001	RV FAC, TAPSE, RV S’
			**GLS −18.5%**	All-cause mortality and HFH (median follow-up of 26 months)	AUC 0.80; sens 76%, spec 71%; *P* < 0.001	
HFpEF	Sibille Lejeune et al. JASE 2020 (30)	149	**GLS −17.5%**	All-cause mortality and first HFH (mean follow-up period of 30 ± 9 months)	HR 2.103; 95% CI 1.237–3.573; *P* = 0.005	RV FAC, TAPSE
Left side heart failure	Mara Gavazzoni et al. EHJ CI, 2020 (31)	458	**FWLS −22.0%**	All-cause mortality and HFH (mean follow-up of 5.4 ± 1.2 years)	AUC 0.68; sens 70%, Spec 65%; *P* < 0.001	
TOF	Ying Gao et al. Front Cardiovasc 2022 (32)	179	**FWLS −17.7%**	All-cause mortality and rehospitalization	C index 0.876, AIC 228 AUC 0.885; sens 87%, Spec 80%	RV FAC
TOF	Cuitlahuac et al. Int J Card image 2022 (33)	34	**FWLS −17%**	Low functional capacity (<7METS)	AUC 0.78; sens 82%, spec 77%	
ARVC	Guido E. Pieles et al. Circ CI, 2019 (34)	120		Diagnosis of ARVC	OR 1.23; 95% CI 1.1–1.37; *P* < 0.01	RV FAC, TAPSE
ARVC	Nitin Malik et al. JAHA, 2020 (35)	40	**FWLS −20.0%**	Structural progression	OR 18.4; 95% CI 2.7–125.8; *P* = 0.003	
Cardiac amyloidosis	Catherina Tjahjadi et al. Am J Cardiol, 2022 (36)	93		All-cause mortality (median follow-up period of 17 months)	HR 0.91; 95% CI 0.86–0.97; *P* = 0.002	
Cardiac amyloidosis	Nowell M Fine et al. Can J Cardiol, 2020 (37)	93		All-cause mortality or CV hospitalization (median follow-up period of 26 months)	HR 1.2 per % change in FWLS; 95% CI 0.8–2.6; *P* < 0.01	
Cardiac sarcoidosis	Cristina Di Stefano et al. BMC, 2020 (38)	83	**GSL −19.9%**	Diagnosis of cardiac sarcoidosis	AUC 0.93; sens 88%,spec 87%	
CRT recipient	Jan Stassen et al. Am J Cardiol, 2022 (39)	871	**FWLS −23.0%**	All-cause mortality (median follow-up of 97 months)	HR 1.618; 95% CI 1.25–2.09; *p* < 0.001	RV FAC, TAPSE
COVID-19	James McErlane et al. Ann Intensive Care, 2022 (40)	94	**FWLS −20.0%**	30-day mortality	HR 2.22; 95% CI 1.14–4.39; *P* = 0.020	
COVID-19	Yuman Li et al. JACC CI, 2020 (41)	120	**FWLS −23.0%**	All-cause mortality (median follow-up period of 51 days)	AUC: 0.87; sens 94% spec 64.7%; *p* < 0.001	RV FAC, TAPSE
ARDS	Jérémie Lemarié et al. Ann Intensive Care, 2020 (42)	48	**GLS −13.70%**	Mortality and cumulative incidence of weaning from MV at day 28	No significant association	

The cut-off value is determined based on ROC curve analysis. Outcomes and analysis result mainly show predictive value for hard outcomes. The conventional parameters described in the column of “Comparison” had shown no significance in multivariable analysis or lower AUC for outcomes compared to RV strain in each study.

RV, right ventricle; STE, Speckle-Tracking echocardiography; HR, hazard ratio; ROC, receiver operating characteristic; AUC, area under the curve; PAH, pulmonary artery hypertension; TR, tricuspid regurgitation; HFpEF, heart failure with preserved ejection fraction; TOF, Tetralogy of Fallot; ARVC, arrhythmogenic right ventricular cardiomyopathy; CRT, cardiac resynchronization therapy; ARDS, acute respiratory distress syndrome; FWLS, right ventricular free wall longitudinal strain; GLS, right ventricular global longitudinal strain; CV, cardiovascular; RVHF, right ventricular heart failure; HFH, heart failure hospitalization; MV, mechanical ventilation; CI, confidence interval; AIC, akaike information criterion; RV FAC, RV fractional area change; RV S’, systolic tissue Doppler velocity of the tricuspid annulus; TAPSE, tricuspid annular plane systolic excursion.

RV LS is typically measured in a modified apical four-chamber view with a frame rate of approximately 50–80 frames per second (fps). The detailed method for appropriate RV strain evaluation has been described by Badano et al. (43). Two methods have been described for RV strain analysis: RV global longitudinal strain (RV GLS), which involves the RV free wall and interventricular septum, and RV free wall longitudinal strain (FWLS), which evaluates only the free wall segments. EACVI/ASE Joint Task Force recommends the use of RV FWLS, excluding the septum, because the interventricular septum includes the left ventricular (LV) component (15). According to the ASE guidelines, RV FWLS <−20% is considered normal (44).

The advantages and disadvantages of the RV LS are illustrated in Figure 1. A major advantage of RV LS is its ability to detect subclinical RV dysfunction earlier than conventional parameters. By tracking speckles, it facilitates a more accurate assessment of RV function. In patients with ARVC, RV LS decreases at the early subclinical stage, whereas RV FAC has shown no differences (45). Haiyan Xu et al. (2021) also reported that RV FWLS is the independent predictor of subclinical chemotherapy-related cardiac dysfunction in patients with breast cancer (46).

The second benefit of RV LS is that it can assess regional RV function. In patients with ARVC, amyloidosis, or other forms of PH, which are known to cause specific segmental issues in the RV, the use of RV strain is particularly valuable (47–49). Since RV strain provides regional strain values, it enables the detection of segmental RV dysfunction including apex and mid of the RV.

In addition, the STE-based strain evaluation has been reported to be less angle-dependent because it does not rely on Doppler alignment. Although the EACVI/ASE Joint Task Force recommendations emphasize analysis in the RV-focused four-chamber view (15), studies have demonstrated that RV strain values from subcostal views are highly correlated with those from the apical view (Pearson's *r* = 0.89) in 94 cases with veno-venous (V-V) ECMO (50). RV strain values from the four-chamber view using transesophageal echocardiography also showed a high correlation with those from the RV-focused four-chamber view using transthoracic echocardiography (*r* = 0.9; 95% CI 0.87–0.94) (51).

On the flip side, the use of RV strain has certain limitations as well. Some reports indicate that RV strain value can be affected by loading conditions (7). Schlangen et al. revealed that the RV LS did not correlate with the RV Ees during preload imposition in humans (6) (Table 1). The load dependency of the LV LS has been demonstrated in human studies (52), and thus, the RV LS is expected to have the same limitation.

Moreover, according to our study on the learning curve for RV strain (53), novice analysts took more time to reach an expert level than LV strain. LV GLS required only 50 studies to achieve American College of Cardiology (ACC)/American Heart Association (AHA)/ASE echocardiography level III competency, whereas RV FWLS required 100 studies for training. One possible explanation for this is the complex RV geometry.

Lastly, though the normal value of RV FWLS was ≤−20% according to ASE, the prognostic cut-off values for each disease differed greatly, as shown in Table 2. The variability in cut-off values for each disease in different studies can add to the complexity in clinical interpretation.

The aspects of RV LS mentioned above are derived solely from data on peak strain values. Beyond peak strain, RV strain analysis offers additional detailed insights into RV function, such as an RV strain curve and strain rate. Badagliacca et al. demonstrated three patterns of strain curves in patients with PAH and their correlation with the prognosis (48). Kirkels et al. reported the clinical utility of the temporal relationship of strain curve patterns and RV mechanical dispersion (MD), which is derived from the standard deviation of the time to peak RV systolic strain (49). As this study demonstrated, the strain curve allows for the assessment of the temporal relationship between strain changes and electrocardiography, which is a unique feature that other conventional parameters do not possess. In strain rate analysis, which measures the velocity of speckle deformation, the early diastolic strain rate (eDSR) serves as a valuable indicator of RV diastolic function, and Chamberlain et al. demonstrated that RV eDSR is a potential predictor of early subclinical post-transplant rejection (54).

In summary, RV strain is capable of detecting mild levels of dysfunction and offers some advantages, including the assessment of RV segments and reduced angle dependency. However, it should be noted that RV strain is load-dependent. Concerning RV strain, significant clinical data have already been accumulated, and thus it will be applied further in clinical settings, potentially replacing conventional parameters.

### 2.3 RV myocardial work

**Figure 5:**
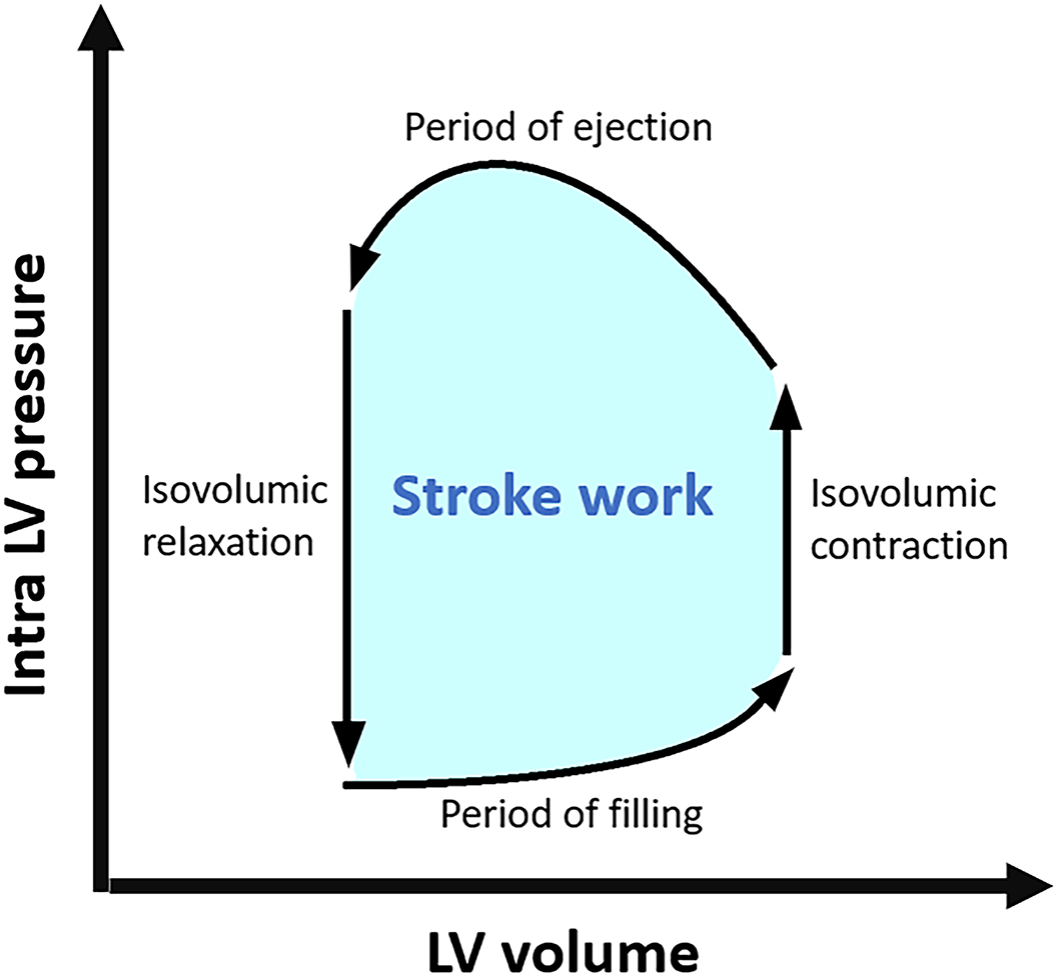
LV pressure-volume loop and stroke work. LV, left ventricular.

Myocardial work (MW) and ventricular pressure-strain loop (PSL) are novel non-invasive surrogates of invasive stroke work (SW) and pressure-volume loop (PVL) derived from conductance catheterization. SW, the area inside the closed PVL, represents the workload of the ventricle to eject stroke volume (SV) to the systemic or pulmonary circulation and can take into account the afterload (Figure 5) (55). In 2012, Russell et al. discovered a good correlation between invasively measured SW and LV MW. In this research, they non-invasively acquired LV PSL and LV MW from the LV longitudinal strain curve by STE and estimated intra-LV pressure waveforms using a branchial artery cuff (56). Furthermore, they demonstrated that non-invasive LV MW accurately correlates with cardiac metabolic oxygen consumption (MVO) using acetate and molecular oxygen positron emission tomography (PET) (56).

MW consists of three main components: global work index (GWI), constructive work (CW), and wasted work (WW). GWI (mmHg%) is the average work performed by the entire ventricle. CW (mmHg%) and WW (mmHg%) are both regional and global measurements, and the ratio of CW to the sum of CW and WW is called cardiac efficiency (CE; %). CE is generally near 100% in a normal healthy myocardium but impairs in pathological conditions such as ischemic cardiomyopathy, heart failure, ventricular dyssynchrony, valvular heart disease (VHD), and hypertrophic and hypertensive cardiomyopathies (55, 57). Edwards NFA et al. (2019) have demonstrated that LV MW indices are superior to LV GLS in predicting significant coronary artery disease by detecting subclinical early ischemic LV dysfunction in patients with normal EF and no regional wall motion abnormalities (58). We previously reported that GWI has the potential to evaluate increased wall stress under different loading conditions in patients with hypertension and dilated cardiomyopathy (59).

**Figure 6:**
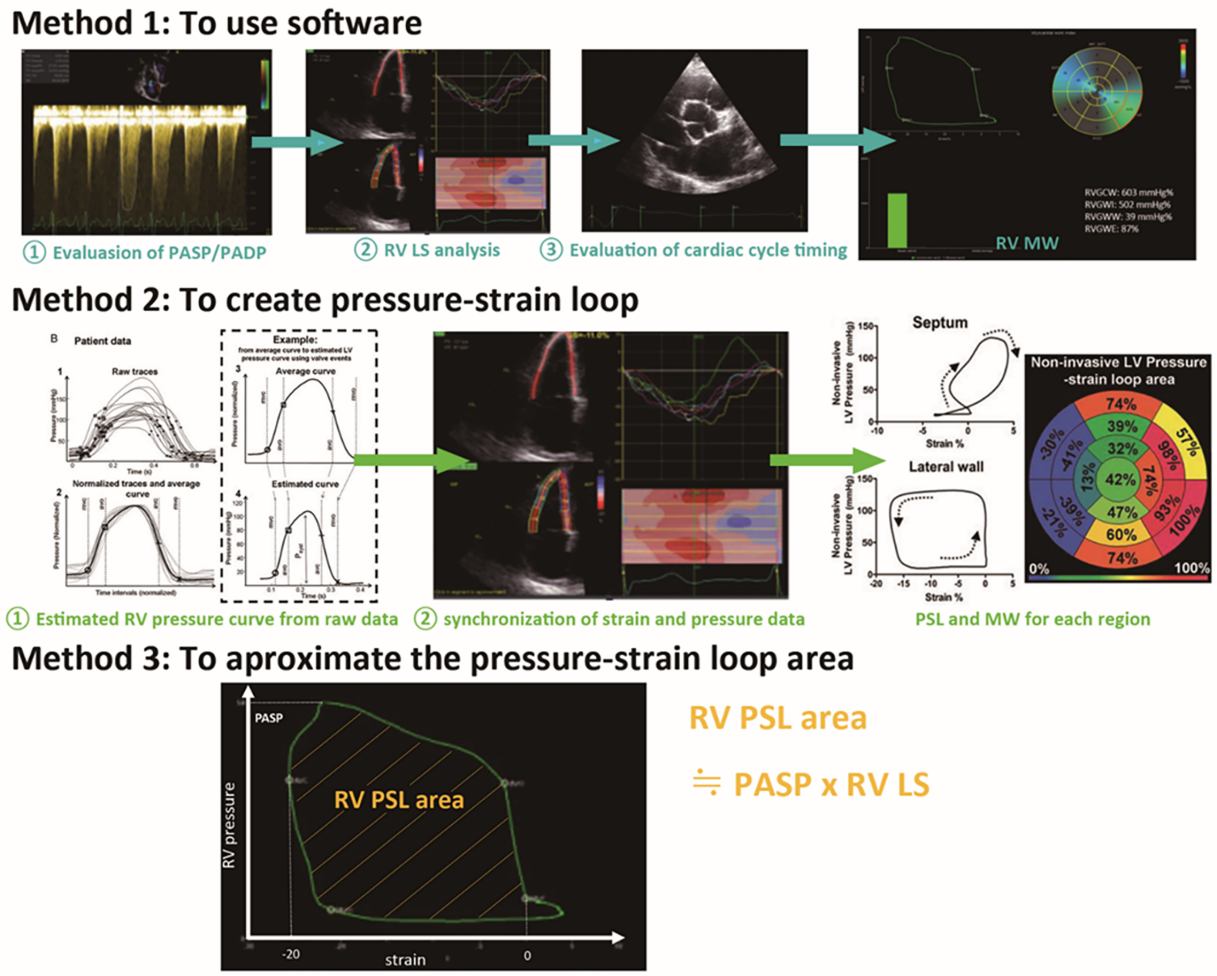
Potential methods to evaluate RV MW by echocardiography. In method 1, the software (EchoPAC; GE Vingmed Ultrasound) for LV MW is applied to RV (11). In method 2, the PSL is created from estimated intra-RV pressure, pulmonary and tricuspid valve opening and closing timing from echocardiography, and strain curve (56). In method 3, approximate RV MW is estimated by the formula PASP × peak RV strain. This is the one we have reported in LV MW. In this approach, we demonstrated a significant correlation between the estimated LV MW called “PSP” and LV SWI (*r* = 0.659, *p* < 0.001) (60). Notably, the research aimed at validating these three approaches for RV MW is very limited and not yet sufficiently validated; thus, further investigations are warranted. RV MW, right ventricular myocardial work; PASP, pulmonary artery systolic pressure; PADP pulmonary artery diastolic pressure; PV, pulmonary valve; TV, tricuspid valve; LV MW, left ventricular myocardial work; RV, right ventricular; PSL, pressure-strain loop; PSP, pressure strain product; SWI, stroke work index. Reprinted with permission from European Heart Journal (56) and European Heart Journal Cardiovascular Imaging (11). ©2012 and 2020, respectively by Oxford University Press via Copyright Clearance Center.

As for LV MW, significant progress has been made in its application to clinical practice. However, data on RV MW remains limited, and its measurement method has yet to be established. Below, we delineate the three potential approaches to obtaining RV MW in Figure 6; (1) apply software (EchoPAC; GE Vingmed Ultrasound) which was originally developed for the LV, to the RV (11). In this approach, tricuspid valve and pulmonary valve opening and closure timing in an electrocardiogram is required; (2) create the PSL from estimated intra RV pressure curve and strain curve (56); and 3) approximate the area of the PSL using the formula PASP × peak RV strain (60).

Although research on RV MW is scarce, some have reported its load independency and clinical utility as detailed in Tables 1, 3.

**Table 3:** Predictive value and a cut-off value of RV myocardial work.

Population	Study	*n*	Parameters used	Component	Outcome/cut-off value	Comparison
PH (Group1,4)	Steele C. Butcher et al. Am J Cardiol, 2022 (10)	51	RVGLS, PASP	RV GCW	All-cause death (HR 1.42 per 100 mm Hg% <900 mm Hg%; 95% CI 1.12–1.81; *P* = 0.004)/RV GCW <550 mmHg% (from spline curve) (KM analysis for all-cause mortality *P* = 0.0007)	RV GLS, RV FAC, TAPSE
				RV GWI	All-cause death (HR 1.46 per 100 mm Hg% <650 mm Hg%, 95% CI 1.09–1.94, *p* = 0.010)/RV GWI <500 mm Hg% (from spline curve) (KM analysis for all-cause mortality *P* = 0.0008)	RV GLS, RV FAC, TAPSE
HTx recipients	Leyla Elif Sade et al. EHJ CI, 2023 (61)	61	RV GLS, Invasive PASP	RV MWI	Rejection-related RV damage in biopsy (HR 0.98; 95% CI 0.97–0.99; *P* = 0.002)/RV MWI <360 mmHg% (ROC analysis for rejection-related RV damage in biopsy AUC 0.812; sens 74% Spec 77%; *P* < 0.001)	RV FWLS
SLE	Xin-Ying Fan et al. Ultrasound Med Biol, 2023 (62)	75	RV GLS PASP, PADP	RV GWW	WHO Function Class ≥2 (AUC 0.893; sens 92% spec 78%)	
PH + healthy cohort	Jian Wang et al. Front Cardiovasc Med, 2022 (63)	79	RVGLS, PASP	RVGWE	All-cause of mortality, hospitalization and need of new specific drug therapy or enhancement on the original therapy basis (OR 0.803; 95% CI 0.698–0.922, *P* = 0.002 / AUC 0.861, *P* < 0.001)	

The column “Parameters used” describes the indices used to obtain myocardial work values, because the approaches to acquire RV myocardial work differed between studies. “Component” shows which components of myocardial work demonstrated the significant predictive value in each study. The conventional parameters described in the column of “Comparison” had shown no significance in multivariable analysis or lower AUC for outcomes compared to RV-PA coupling in each study.

RV, right ventricular; PH, pulmonary hypertension; HFrEF, heart failure with reduced ejection fraction; HTx, heart transplantation; SLE, systemic lupus erythematosus; RVGLS, right ventricular global longitudinal strain; PASP, pulmonary artery systolic pressure; PADP, pulmonary artery diastolic pressure; GCW, global constructive work; GWI, global work index; MWI, myocardial work index; GWW, global wasted work; GWE, global work efficiency; HR, hazard ratio; CI, confidence interval; KM, Kaplan-Meier; ROC, receiver operating characteristic; AUC, area under the curve; OR, odds ratio; GLS, right ventricular global longitudinal strain; RV FAC, RV fractional area change; TAPSE, tricuspid annular plane systolic excursion; FWLS, right ventricular free wall longitudinal strain.

Butcher et al. revealed that non-invasive RV global CW correlated more closely with invasively measured stroke volume and stroke volume index in 22 cardiac patients with heart failure and reduced ejection fraction (*r* = 0.63 and *r* = 0.59, respectively), compared to the standard echo parameters (TAPSE, RVFAC, RVFWLS, RV GLS) (11).

Sade et al. reported that the RV MW index was the strongest predictor of rejection revealed by endomyocardial biopsy in 61 heart transplant recipients [area under curve (AUC): 0.812, 95% CI:0.69–0.94] compared to T1 time and extracellular volume in cardiac magnetic resonance imaging (61). This can be a potential novel assessment in the follow-up of heart transplant recipients to non-invasively detect subclinical RV dysfunction due to rejection.

Additionally, RV MW has demonstrated excellent interobserver reproducibility comparable to that of RV GLS. Butcher et al. reported significant inter-analyst reproducibility [ICC 0.915 for RVGCW (*P* < 0.001)] and excellent intra-observer reproducibility [ICC 0.938 for RVGWW (*P* < 0.001)] (11). This remarkable variability has been reported in another study as well (62).

RV MW has limitations as well. Firstly, correct non-invasive estimation of RV intracardiac pressure is theoretically difficult. In MW analysis, we need intraventricular pressure as an afterload indicator. In LV analysis, brachial systolic blood pressure is used to estimate the intra-LV pressure. However, the estimation of the intra-RV pressure is challenging. The reason for this is that echocardiographic estimation of PASP and pulmonary artery diastolic pressure (PADP) can occasionally be impossible or inaccurate, particularly in cases without TR and pulmonary regurgitation, or with severe TR.

Secondly, the RV MW by PSL does not consider RV geometry. The afterload on the RV depends only on the RV pressure parameter in this evaluation, and strictly speaking, we need to consider the radius and wall thickness as a part of the afterload, which is governed by Laplace's law: RV wall stress (dynes/cm^2^) = (RV intra-pressure × radius)/2 × LV wall thickness (55).

In summary, MW is a new parameter, and its accessibility remains limited owing to the lack of established measurement methods and intricate principles. However, MW possesses unique characteristics superior to conventional indices, including the capability to assess MVO, CE, and regional CW. Further investigations will drive clinical application for more precise routine assessment of RV dysfunction.

### 2.4 RV-PA coupling

“RV- pulmonary artery (PA) coupling” is an assessment of the RV contractile capacity with consideration of the afterload, similar to RV MW. The gold standard for measuring RV-PA coupling is the “Ees/Ea” ratio, originally obtained invasively using a conductance catheter. A normal Ees/Ea ratio indicates that RV contractility works effectively under the afterload exerted on the RV and generates an appropriate stroke volume. Conversely, a decreased Ees/Ea ratio signifies “uncoupling”, where RV contractility diminishes under the existing afterload conditions and is unable to supply the necessary stroke volume to pulmonary circulation (64). Although RV is tolerant to changes in preload, its function can be easily impaired by a slight increase in afterload as mentioned in the introduction (3); thus, considering afterload is crucial when evaluating RV function.

Lately, several novel echocardiographic indices have been tested as non-invasive surrogates of RV-PA coupling; TAPSE/PASP, RV S'/PASP, RVFAC/PASP, and RV LS/PASP, all of which represent the ratio of contractile parameters to PASP. As these parameters have shown a higher prognostic ability in clinical research (Table 4), they have gradually been utilized in clinical settings as novel load-independent parameters. Most recently, the 2022 European Society of Cardiology (ESC)/European Respiratory Society (ERS) guidelines for PH have recommended TAPSE/PASP as an indicator of risk stratification, using cut-off values of 0.19 mm/mmHg and 0.32 mm/mmHg for intermediate and high risk, respectively (78).

**Table 4:** Predictive value and cut-off value of RV-PA coupling by echocardiography.

Population	Study	*n*	Cut-off value	Multivariable (HR/OR) or ROC (AUC) analysis result	Comparison
Pre capillary PAH	Serkan Ünlü et al. EHJ CI, 2023 (65)	65	RV FWLS/PASP	Death or heart/lung transplantation (10 years) (HR 6.99; 95% CI 3.71–13.15; *P* < 0.001)	RV FWLS, TAPSE, TAPSE/PASP, RV FAC/PASP, RV FAC
			RV FWLS/PASP 0.19%/mmHg	Death or heart/lung transplantation (10 years) (AUC 0.9 a sensitivity of 92% and specificity of 82.5%)	
SLE PAH	Xiaoxiao Guo et al. EHJ CI 2021 (66)	112	TAPSE/PASP 0.184 mm/mmHg	All-cause mortality and clinical worsening (HR 2.77, 95% CI 1.55–4.93, *P *=* *0.001)	
				All-cause mortality and clinical worsening (HR 0.74, specificity 78%, sensitivity 62%, *P *<* *0.001)	
PAH or CTEPH	Khodr Tello et al. Circ CI, 2019 (13)		TAPSE/PASP 0.31 mm/mmHg	Overall mortality (HR hazard ratio: 1.85; 95% CI, 1.16–2.95)	
PE	Mads D Lyhne et al. EHJ CI, 2021 (67)	627	TAPSE/PASP	7-day composite outcome of death or haemodynamic deterioration (OR 0.028; 95% CI 0.010–0.087; *P* < 0.0001)	
			TAPSE/PASP 0.387 mm/mmHg	7-day composite outcome of death or haemodynamic deterioration (AUC 0.740; 95% CI 0.694–0.787)	
TAVI	Catalina A Parasca et al. Front Cardiovasc Med, 2023 (68)	160	RV FWLS/PASP 0.63%/mmHg	3 year mortality (AUC 0.65, *p* = 0.001; sensitivity 86%, specificity 57%)	RV GLS/PASP, TAPSE/PASP, RV S’/PASP, RV FAC/PASP
				MACE (HR = 4.14, CI = 1.37–12.5, *p* = 0.012)	
TAVI or SAVR (PARTNER3 Trial)	Thomas J Cahill et al. JACC Cardiovasc Interv, 2022 (69)	570	TAPSE/PASP 0.55 mm/mmHg	All-cause mortality, stroke, and rehospitalization at the 2-year follow-up (HR: 1.92; 95% CI: 1.04–3.57; *P* = 0.038)	TAPSE, RV S’,
TMVr	Nicole Karam et al. JACC CI 2021 (70)	817	TAPSE/PASP 0.274 mm/mm Hg	Survival rate at 2 years (OR 1.62; 95% CI 1.14–2.31; *p* = 0.007)	
TTVr or TTVR	Michael l Brenner et al. JACC 2022 (71)	444	TAPSE/PASP 0.406 mm/mmHg	All-cause mortality (OR 0.42; 95% CI 0.19–0.93; *P* = 0.032)	
TR	Federico Fortuni et al. Am J Cardiol, 2021 (72)	1,149	TAPSE/PASP 0.31 mm/mmHg	All-cause mortality (median follow-up of 51 months) (HR 1.462; 95% CI 1.192–1.793; *p* < 0.001)	
CRT recipients	Jan Stassen et al. ESC Heart Fail, 2022 (39)	807	TAPSE/PASP 0.45 mm/mmHg	All-cause mortality (median follow-up 97 months) (HR 1.437; 95% CI 1.145–1.805; *P* = 0.002)	TAPSE
HFrEF	Mohamed Naseem et al. BMC, 2022 (73)	200	TAPSE/PASP	In-hospital mortality (OR=18.813; 95% CI, 1.974–179.275, *p*-value = 0.011)	
			TAPSE/PASP 0.4 mm/mmHg	In-hospital mortality (AUC 0.666; sens 79.17, spec 47.73)	
HFrEF	Alexander Schemeisser et al. EHJ CI, 2021 (12)	110	TAPSE/PASP 0.38 mm/mmHg	All-cause mortality (AUC = 0.709, *P* = 0.001) (HR 0.07; 95% CI 0.005–0.920; *P* = 0.043)	
COVID-19	Francesca Bursi et al. JAHA, 2022 (74)	133	TAPSE/PASP 0.57 mm/mm Hg	In-hospital death (75% sensitivity and 70% specificity)	
				In-hospital death HR, 4.8 [95% CI, 1.7–13.1]; *P* = 0.007	
COVID-19	Michele D'Alto et al. Crit Care, 2020 (75)	94	TAPSE/PASP	All-cause mortality (HR 0.988 0.977–0.998 *P* 0.018)	
V-A ECMO weaning	Darae Kim et al. JACC CI, 2021 (76)	79	RV S’/PASP 0.33	Successful weaning from V-A ECMO (AUC 0.695; 95% CI 0.581–0.793; *p* = 0.002)	Conventional echo criteria (LVEF >20%, VTI ≥10 cm, MV annulus S ≥6 cm/s)
			RV FWLS/PASP 0.45%/mmHg	Successful weaning from VA-ECMO (AUC 0.681; 95% CI 0.567–0.782; *p* = 0.004)	
CICU (ACS, HF, cardiogenic shock)	Jacob C Jentwer et al. JAHA, 2021 (77)	4,259	RV S’/PASP	• In-hospital mortality (adjusted unit OR, 0.68 per each 0.1-unit higher ratio; 95% CI, 0.58–0.79; *P* < 0.001) • 1-year mortality (adjusted unit HR, 0.83 per each 0.1-unit higher ratio; 95% CI, 0.77–0.90; *P* < 0.001)	

Cut-off value is determined based on ROC curve analysis. The conventional parameters described in the column of “Comparison” had shown no significance in multivariable analysis or lower AUC for outcomes compared to RV-PA coupling in each study.

RV, right ventricle; PA, pulmonary artery; HR, hazard ratio; OR, odds ratio; ROC, receiver operating characteristic; AUC, area under the curve; PAH, pulmonary artery hypertension; SLE, systemic lupus erythematosus; CTEPH, chronic thromboembolic pulmonary hypertension; PE, pulmonary embolism; TAVI, transcatheter aortic valve replacement; surgical aortic valve replacement; TMVr, transcatheter mitral valve repair; TTVr, transcatheter tricuspid valve repair; TTVR, transcatheter tricuspid valve replacement; TR, tricuspid regurgitation; HFrEF, heart failure with reduced ejection fraction; VA ECMO, veno-arterial extracorporeal membrane oxygenation; CICU, cardiac intensive care unit; ACS, acute coronary syndrome; HF, heart failure; FWLS, right ventricular free wall longitudinal strain; PASP, pulmonary artery systolic pressure; TAPSE, tricuspid annular plane systolic excursion; RV S’, systolic tissue Doppler velocity of the tricuspid annulus; CI, confidence interval; MACE, major adverse cardiac event; RV FAC, RV fractional area change; GLS, right ventricular global longitudinal strain; LVEF, left ventricular ejection fraction; VTI, velocity time integral; MV, mitral valve.

One aspect to note with this parameter is that the gold standard of RV-PA coupling, Ees/Ea, is originally expressed as Ees = PASP/end-systolic volume (ESV) and Ea = PASP/SV, which simplifies to Ees/Ea = SV/ESV, not TAPSE/PASP, RV S'/PASP, RVFAC/PASP, nor RV LS/PASP. Previous studies have shown that both TAPSE/PASP and SV/ESV are correlated to a certain extent with invasive Ees/Ea (Table 1).

The RV-PA coupling indices obtained by echocardiography have several merits, as shown in Figure 1. First of all, these parameters have been demonstrated to significantly correlate with the invasive parameter, Ees/Ea (Table 1), and can be described as load-independent. Secondly, the notable difference with other novel parameters is that specific software or advanced measurement methods are not required. Its value can be readily calculated as long as M-mode and continuous-wave Doppler measurements are available. These aspects make echocardiographic RV-PA coupling versatile and applicable to a wide range of clinical settings including emergency room and ICU (74–76).

On the other hand, echocardiographic RV-PA coupling has several limitations based on the weaknesses of its components: TAPSE, RV S', RV FAC, and PASP. For instance, TAPSE and RV S' are angle-dependent and can be overestimated in inappropriate images as previously discussed, and PASP is challenging to estimate in cases without TR. Furthermore, TAPSE/PASP does not consider the volume load, and a change in preload on RV has been reported to affect the value of TAPSE/PASP in animal experiments (79).

Along with RV MW, echocardiographic RV-PA coupling has load independency and a promising predictive value for clinical outcomes. Notably, since echocardiographic RV-PA coupling is easier to obtain than the other two novel parameters and significant clinical evidence has been already accumulated, it has the potential to be applied in a wider range of clinical situations.

### 2.5 Novel RV function assessment in different pathologies

In this section, we list representative diseases causing RV dysfunction for which data on novel echocardiographic parameters have been demonstrated and outline these clinical values.

#### 2.5.1 Pulmonary arterial hypertension (PAH)

PAH can be caused by various conditions, including heritable, collagen diseases, and toxins. In patients with PAH, vasculopathy due to underlying causes leads to increased pulmonary vein resistance (PVR), which can be a burden on the RV as afterload (78). Increased afterload causes a decline in RV contractility, eventually leading to RV failure, which is the main reason for the worse outcomes in PAH (78). PAH affects approximately 48–55 cases/million adults, and the short-term mortality of patients hospitalized with PAH and concomitant RV failure is as high as 40% (78, 80). Therefore, it is crucial to accurately capture the RV function with the incorporation of afterload. We outline a few studies below.

Wright et al. reported that baseline RV FWLS and a change in RV FWLS were significantly associated with all-cause mortality (HR 0.90; 95% CI 0.83–0.97), independent of PASP, RV FAC, and TAPSE in 96 patients with PAH (24). Ünlü et al. (2023) reported that TAPSE/PASP, RV FAC/PASP, and RV FWLS/PASP were lower in those with worse clinical outcomes, whereas those with PASP obtained by catheterization, TAPSE and RV FAC showed no significant difference (65).

In cases with PAH, load-incorporating indices such as TAPSE/PASP and RV MW will be especially beneficial for diagnosis and treatment.

#### 2.5.2 Valvular heart disease (VHD)

RV dysfunction progresses as VHD severity increases, primarily due to both volume and pressure loading on RV. Timely intervention, before RV dysfunction becomes irreversible, is essential for them (81, 82). The utility of novel echocardiographic parameters has been demonstrated in cases of aortic stenosis (AS), mitral regurgitation (MR), and TR as below.

AS causes volume and pressure overload on RV, and 48%–75% of severe AS cases are reported to have PH (83). Among patients undergoing TAVI or surgical aortic valve replacement, Cahill et al. revealed that TAPSE/PASP has better predictive value for clinical outcomes (HR: 1.92; 95% CI: 1.04–3.57), than TAPSE and RV S' (69).

MR is a common VHD inducing combined pre- and post-capillary PH and 59.5% of patients with moderate to severe MR are accompanied by PH (84). In patients after transcatheter mitral valve repair, Karam et al. demonstrated that TAPSE/PASP can independently predict 2-year survival (OR 1.62; 95% CI 1.14–2.31) and adding impaired coupling to the standard risk stratification model has provided an incremental value to mortality prediction which conventional parameters have not (70).

TR is a right-sided VHD and is more directly and frequently associated with RV dysfunction than left-heart VHDs. Furthermore, the effect of TR on the RV is complex and differs from that of other VHDs, since TR reduces the afterload on the RV, leading to the RV appearing to function well or normally. However, RV dysfunction is particularly critical in this cohort, because treatment for TR can paradoxically increase the afterload on the RV and delayed therapeutic intervention in cases of significant RV dysfunction can lead to tragic RV failure following treatment (81, 82). Nevertheless, an absolute parameter of RV function to guide intervention has not yet been established. Recently, Hinoja et al. reported that RV FWLS >−21.5% has a more significant prognostic value for all-cause mortality and heart failure hospitalization (AUC 0.82; sensitivity 80%, specificity 74%) than conventional parameters (TAPSE, RV S', RV FAC) in patients with TR (29). Besides that, Brener et al. showed that TAPSE/PASP can significantly predict all-cause mortality (OR 0.42; 95% CI 0.19–0.93) in patients after transcatheter tricuspid valve interventions (71).

All three common VHDs can change loading conditions and are frequently accompanied by RV failure. Thus, novel RV parameters can be helpful in guiding follow-up and in judging the timing of treatment.

#### 2.5.3 Cardiomyopathy

Cardiomyopathy directly damages the RV myocardium and impairs RV contractility due to various causes. Since some of their damages are irreversible due to lack of effective therapy, delayed recognition can cause refractory heart failure and arrhythmia. In this cohort, the characteristic pattern of regional wall dysfunction gained by RV strain is also beneficial for diagnosis and determination of severity (30). We discuss three representative cardiomyopathies that can affect RV function; ARVC, cardiac sarcoidosis, and cardiac amyloidosis.

ARVC, the inherited RV cardiomyopathy, causes RV dysfunction earlier than LV dysfunction because replacement by fibrofatty tissue in desmosome proteins initially occurs in the thinner wall. As ARVC is characterized by life-threatening ventricular arrhythmias in healthy young individuals, early diagnosis through the detection of slight RV dysfunction is important. As detailed in the section of RV strain, strain curve patterns on basal RV segment and RV mechanical dispersions are reliable indicators for assessing subclinical stage and future risk (49).

Sarcoidosis, a systemic granulomatous disease, can affect the myocardium as well. In patients with cardiac sarcoidosis, RV dysfunction advances with the impairment of LV function. Stefano et al. have reported that RV GLS is useful in the diagnosis of cardiac sarcoidosis (cut-off value −19.9%; AUC 0.93; sensitivity 88%, specificity 87%) and is well correlated with adverse cardiac events (38).

In amyloidosis, abnormal proteins called amyloids can infiltrate the myocardium. Functional impairment and thickening are observed in the RV as well. In cardiac amyloidosis, specific regional impairment in both LV and RV, called “apical sparing”, in which the impairment initiates from the basal ventricular area, is observed. Moñivas et al. demonstrated that RV apical ratio (=average of apical strain/average of basal strain + average of mid strain) is significantly higher in patients with systemic light-chain cardiac amyloidosis, compared to control (47).

Since cardiomyopathies exhibit specific RV regional dysfunctions, and early diagnosis is critical for their prognosis, detailed analysis using RV strain is effective for both diagnosis and follow-up.

#### 2.5.4 Pulmonary embolism (PE)

Acute pulmonary embolism (PE) can cause a sudden increase in RV afterload. It often exceeds RV contractile capacity and causes RV dysfunction. PE remains the third leading cause of cardiovascular mortality, and RV failure is their most common cause of death (85). Thus, proper assessment of RV dysfunction is crucial for predicting the clinical course and determining the timing of therapeutic interventions, such as thrombolysis, veno-arterial extracorporeal membrane oxygenation (V-A ECMO), and thrombectomy.

Lyhne et al. demonstrated that TAPSE/PASP was significantly associated with 7- and 30-day all-cause mortality in 627 patients with PE, whereas conventional parameters such as TAPSE and PASP were not (67). Kiamanesh et al. reported that TAPSE/RVSP was one of the independent predictors of adverse PE-related events compared with other risk stratification methods, including computed tomography-derived RV dysfunction and the Bova score in normotensive PE (86).

Since consideration of afterload is important for the assessment of RV function in PE, the TAPSE/PASP ratio is a useful parameter that can be readily obtained even in the intensive care unit (ICU).

#### 2.5.5 Coronavirus disease 2019 (COVID-19)

COVID-19 caused a global pandemic, resulting in significant morbidity and mortality. COVID-19 can be complicated by acute respiratory distress syndrome (ARDS) and PE, both of which can increase RV afterload, and systemic inflammation by the virus can injure RV as well (42).

Francesca et al. reported that TAPSE/PASP had significant predictive value for in-hospital death in 133 patients with COVID-19 (74). Li Y et al. revealed that RV LS had more accurate ability to predict all-cause mortality than TAPSE and RV FAC in 120 patients with COVID-19. Furthermore, the cut-off value of −23.0% had an excellent predictive capability for the outcome (AUC: 0.87; sensitivity 94% specificity 64.7%) (41).

In patients with COVID-19, invasive mechanical ventilation increases PASP and RV afterload (40). Thus, the load-considering feature of the novel parameters is helpful. Additionally, in ICU settings, the angle independence of RV strain is supportive in acquiring echocardiographic images, even in patients with high positive end-expiratory pressure, in whom only the subcostal approach may be useful to obtain images.

#### 2.5.6 ECMO

V-A and V-V ECMO are generally used to treat cardiogenic shock and respiratory failure, respectively. V-A ECMO relieves the RV by decreasing the preload, but it can increase pressure in the aorta, possibly increasing left-sided heart pressure, pulmonary artery pressure, and consequently, RV afterload. On the flip side, V-V ECMO can deliver highly oxygenated blood to the lung circulation, reduce PVR, and decrease RV afterload. Since both ECMO configurations support RV, unnoticed RV dysfunction becomes evident after ECMO decannulation and cessation of RV support (87). Therefore, assessing RV function incorporating complicated loading conditions during ECMO therapy is essential to prevent RVF following ECMO removal.

Kim et al. reported that RV S'/PASP >0.35 (AUC 0.695; 95% CI 0.581–0.793) or RV FWLS/PASP >0.45 (AUC 0.681; 95% CI 0.567–0.782) were more reliable to predict successful weaning in V-A ECMO than the conventional echo criteria (LVEF >20%, velocity time integral ≥10 cm, MV annulus S' ≥6 cm/s) (76). Gambaro et al. demonstrated that RV FWLS could be useful in predicting adverse events 30 days after V-A ECMO decannulation (88).

Notably, studies have shown that novel RV echocardiographic parameters are useful as an indicator for V-A ECMO weaning, as much as LV parameters.

## 3 Conclusion

In this review, we have outlined characteristics of three new echocardiographic indices for RV function assessment: RV strain, RV MW, and RV-PA coupling.

Of the three parameters, RV strain has already accumulated the most clinical data. It enables RV segmental assessment, angle independence, and better reproductivity compared to conventional parameters, and thus will be more applied in clinical settings. However, the load dependency is reported as a limitation as well. On the other hand, RV MW and RV-PA coupling are parameters that incorporate afterload. RV MW has the distinct capability to assess MVO, regional wasted work, and regional work efficiency, but the data on RV MW are sparse, and its clinical application is still highly limited compared to the other two parameters. Echocardiographic RV-PA coupling is relatively easier to obtain than the other two novel parameters, and clinical evidence has been accumulated as well in various cohorts.

All these have the potential to become key parameters in RV function assessment in the near future, and larger-scale studies and further preclinical fundamental investigations will enhance their prospects.
