# Supplementary figures and images for: Current trends and latest developments in echocardiographic assessment of right ventricular function: load dependency perspective
Source: Front Cardiovasc Med. 2024 Jul 1;11:1365798. doi: 10.3389/fcvm.2024.1365798 (PMC11249019; doi:10.3389/fcvm.2024.1365798)

Supplementary Figure 1. Flow diagram

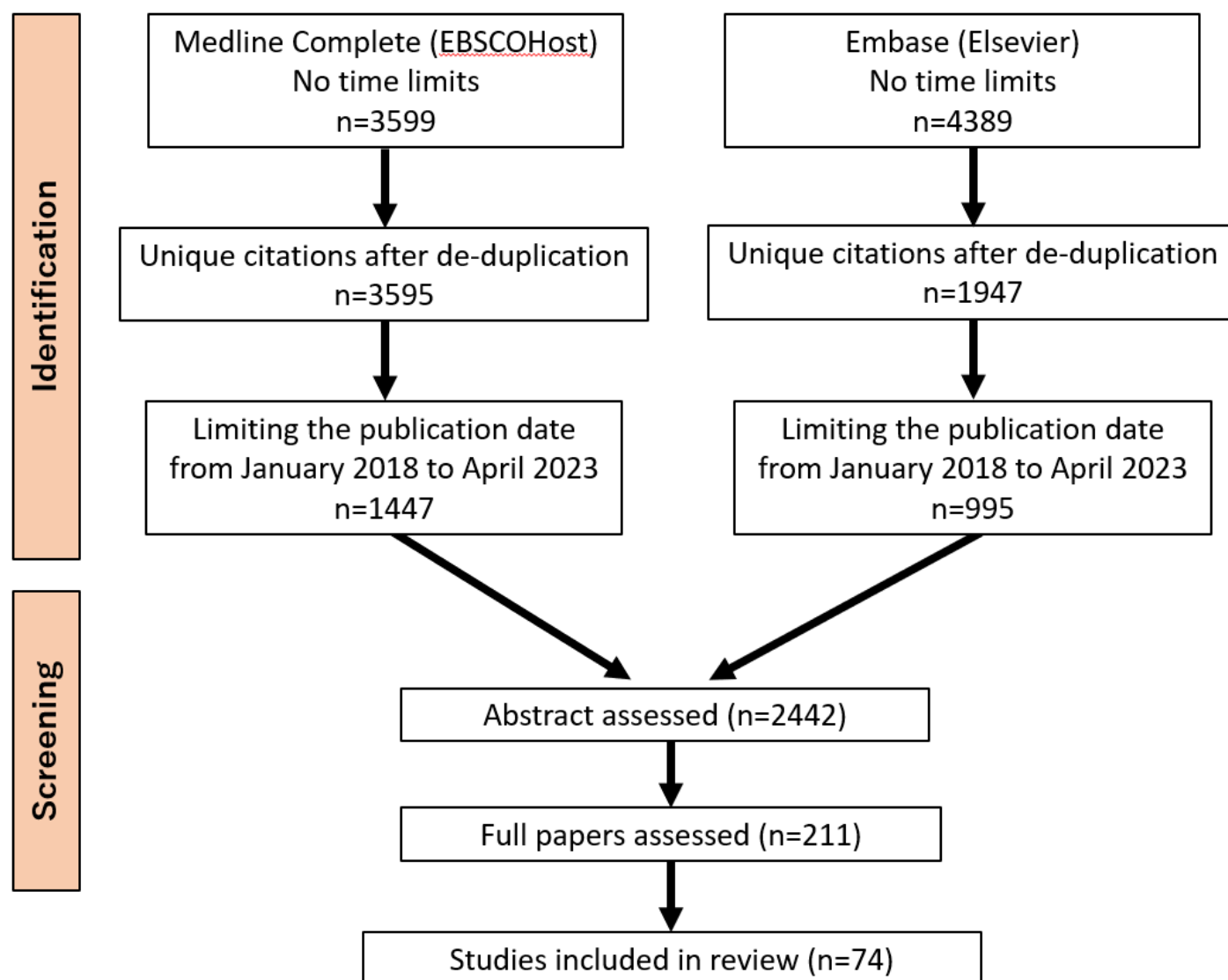

Supplement: Supplementary file 1 [file Image1.pdf]
