# Supplementary material for: Current trends and latest developments in echocardiographic assessment of right ventricular function: load dependency perspective
Source: Front Cardiovasc Med. 2024 Jul 1;11:1365798. doi: 10.3389/fcvm.2024.1365798 (PMC11249019; doi:10.3389/fcvm.2024.1365798)
Supplement: Supplementary file 2 [file Table1.pdf]

Supplementary Table 1.
Search strategies – novel echo parameters for right ventricular function

Create and run by Jana Waldmann on 16 May 2023

MEDLINE Complete (EbscoHOST)

#	Query	Limiters/Expanders	Results
S17	S14 NOT S15	Limiters - Date of Publication: 20170101-; English Language Expanders - Apply related words; Apply equivalent subjects Search modes - Find all my search terms	1683
S17	S14 NOT S15	Limiters - English Language Expanders - Apply related words; Apply equivalent subjects Search modes - Find all my search terms	3,599
S16	S14 NOT S15	Expanders - Apply related words; Apply equivalent subjects Search modes - Find all my search terms	3,762
S15	PT (comment) OR PT (commentary) OR PT (conference) OR PT (congress) OR PT (editorial) OR PT (letter)	Expanders - Apply related words; Apply equivalent subjects Search modes - Find all my search terms	2,219,983
S14	S3 AND S10 AND S13	Expanders - Apply related words; Apply equivalent subjects Search modes - Find all my search terms	3,816
S13	S11 OR S12	Expanders - Apply related words; Apply equivalent subjects Search modes - Find all my search terms	24,824
S12	TI (("right ventric*" OR "right heart" OR "right cardiac") N2 (function* OR dysfunction* OR failure)) OR AB (("right ventric*" OR "right heart" OR "right cardiac") N2 (function* OR dysfunction* OR failure))	Expanders - Apply related words; Apply equivalent subjects Search modes - Find all my search terms	18,149

S11	(MH "Ventricular Function, Right") OR (MH "Ventricular Dysfunction, Right")	Expanders - Apply related words; Apply equivalent subjects Search modes - Find all my search terms	13,150
S10	S4 OR S5 OR S6 OR S7 OR S8 OR S9	Expanders - Apply related words; Apply equivalent subjects Search modes - Find all my search terms	941,150
S9	(MH "Stroke Volume")	Expanders - Apply related words; Apply equivalent subjects Search modes - Find all my search terms	51,886
S8	TI ((novel OR new) N2 paramet*) OR AB ((novel OR new) N2 paramet*)	Expanders - Apply related words; Apply equivalent subjects Search modes - Find all my search terms	11,266
S7	TI ("s'/pasp" OR "s'/rvsp" OR "s'/trpg" OR (("tricuspid annular systolic" OR "ta systolic") N3 ("pulmonary artery systolic pressure" OR pasp OR "tricuspid regurgitation pressure gradient" OR trpg OR "right ventricular systolic pressure" OR "right ventricle systolic pressure" OR "rv systolic pressure" OR rvsp))) OR AB ("s'/pasp" OR "s'/rvsp" OR "s'/trpg" OR (("tricuspid annular systolic" OR "ta systolic") N3 ("pulmonary artery systolic pressure" OR pasp OR "tricuspid regurgitation pressure gradient" OR trpg OR "right ventricular systolic pressure" OR "right ventricle systolic pressure" OR "rv systolic pressure" OR rvsp)))	Expanders - Apply related words; Apply equivalent subjects Search modes - Find all my search terms	6
S6	TI (("sv" OR "stroke volume") N5 ("esv" OR "end-systolic volume")) OR AB (("sv" OR "stroke volume") N5 ("esv" OR "end-systolic volume"))	Expanders - Apply related words; Apply equivalent subjects Search modes - Find all my search terms	628
S5	TI ((tapse OR "tricuspid annular plane systolic excursion" OR rvfac OR "right ventricular fractional area change" OR "right ventricle fractional area change" OR "rv fractional area change" OR rvfws OR "right ventricular free wall strain" OR "right ventricle free wall strain" OR "rv free wall strain" OR rvgls OR "right ventricular global longitudinal strain" OR "right ventricle global longitudinal strain" OR "rv global longitudinal strain") N5 (pasp OR "pulmonary artery systolic pressure" OR rvsp OR "right ventricular systolic pressure" OR "right ventricle systolic pressure" OR "rv systolic pressure" OR "rv systolic pressure" OR trpg OR "tricuspid regurgitation pressure gradient")) OR AB ((tapse OR "tricuspid annular plane systolic excursion" OR rvfac OR "right ventricular fractional area change" OR "right ventricle fractional area change" OR "rv fractional area change" OR rvfws OR "right ventricular free wall strain" OR "right ventricle free wall strain" OR "rv free wall strain" OR rvgls OR "right ventricular global longitudinal strain" OR "right ventricle	Expanders - Apply related words; Apply equivalent subjects Search modes - Find all my search terms	212

	global longitudinal strain" OR "rv global longitudinal strain") N5 (pasp OR "pulmonary artery systolic pressure" OR rvsp OR "right ventricular systolic pressure" OR "right ventricle systolic pressure" OR "rv systolic pressure" OR "rv systolic pressure" OR trpg OR "tricuspid regurgitation pressure gradient"))		
S4	TI ("ejection fraction" OR "ef" OR "rvef" OR strain OR "speckle tracking" OR "longitudinal strain" OR "gls" OR "free wall strain" OR "fs" OR "fws" OR "pressure strain loop" OR "myocardial work" OR "mw" OR "stroke work" OR "sw" OR "right ventricular pulmonary artery coupling" OR "right ventricle pulmonary artery coupling" OR "rv-pa coupling" OR "rvpa coupling") OR AB ("ejection fraction" OR "rvef" OR strain OR "speckle tracking" OR "longitudinal strain" OR "gls" OR "free wall strain" OR "pressure strain loop" OR "myocardial work" OR "stroke work" OR "right ventricular pulmonary artery coupling" OR "right ventricle pulmonary artery coupling" OR "rv-pa coupling" OR "rvpa coupling")	Expanders - Apply related words; Apply equivalent subjects Search modes - Find all my search terms	910,535
S3	S1 OR S2	Expanders - Apply related words; Apply equivalent subjects Search modes - Find all my search terms	228,141
S2	TI (echocardiogr* OR ((heart OR cardiac) N2 (echogra* OR ultrasound* OR "ultra sound*" OR ultrasonogra* OR "ultra sonogra*" OR sonogra* OR ultrasonic OR "ultra sonic"))) OR AB (echocardiogr* OR ((heart OR cardiac) N2 (echogra* OR ultrasound* OR "ultra sound*" OR ultrasonogra* OR "ultra sonogra*" OR sonogra* OR ultrasonic OR "ultra sonic")))	Expanders - Apply related words; Apply equivalent subjects Search modes - Find all my search terms	177,769
S1	(MH "Echocardiography+")	Expanders - Apply related words; Apply equivalent subjects Search modes - Find all my search terms	148,705

Embase (Elsevier)

No.	Query	Results
#17	#3 AND #11 AND #14 AND ([article]/lim OR [article in press]/lim OR [review]/lim) AND [english]/lim AND [2017-2023]/py	2333
#16	#3 AND #11 AND #14 AND ([article]/lim OR [article in press]/lim OR [review]/lim) AND [english]/lim	4389
#15	#3 AND #11 AND #14	9173
#14	#12 OR #13	36901
#13	(('right ventric*' OR 'right heart' OR 'right cardiac') NEAR/2 (function* OR dysfunction* OR failure)):ti,ab,kw	32288
#12	'heart right ventricle function'/de	10088
#11	#4 OR #5 OR #6 OR #7 OR #8 OR #9 OR #10	873355
#10	((novel OR new) NEAR/2 paramet*):ti,ab,kw	11953

#9	's/pasp':ti,ab,kw OR 's/rvsp':ti,ab,kw OR 's/trpg':ti,ab,kw OR ((('tricuspid annular systolic' OR 'ta systolic') NEAR/3 ('pulmonary artery systolic pressure' OR pasp OR 'tricuspid regurgitation pressure gradient' OR trpg OR 'right ventricular systolic pressure' OR 'right ventricle systolic pressure' OR 'rv systolic pressure' OR rvsp)):ti,ab,kw)	32
#8	(('sv' OR 'stroke volume') NEAR/5 ('esv' OR 'end-systolic volume')):ti,ab,kw	1095
#7	((tapse OR 'tricuspid annular plane systolic excursion' OR rvfac OR 'right ventricular fractional area change' OR 'right ventricle fractional area change' OR 'rv fractional area change' OR rvfws OR 'right ventricular free wall strain' OR 'right ventricle free wall strain' OR 'rv free wall strain' OR rvgls OR 'right ventricular global longitudinal strain' OR 'right ventricle global longitudinal strain' OR 'rv global longitudinal strain') NEAR/5 (pasp OR 'pulmonary artery systolic pressure' OR rvsp OR 'right ventricular systolic pressure' OR 'right ventricle systolic pressure' OR 'rv systolic pressure' OR 'rv systolic pressure' OR trpg OR 'tricuspid regurgitation pressure gradient')):ti,ab,kw	621
#6	'ejection fraction':ti,ab,kw OR 'ef':ti OR 'rvef':ti,ab,kw OR strain:ti,ab,kw OR 'speckle tracking':ti,ab,kw OR 'longitudinal strain':ti,ab,kw OR 'gls':ti,ab,kw OR 'free wall strain':ti,ab,kw OR 'fs':ti OR 'fws':ti OR 'pressure strain loop':ti,ab,kw OR 'myocardial work':ti,ab,kw OR 'mw':ti OR 'stroke work':ti,ab,kw OR 'sw':ti OR 'right ventricular pulmonary artery coupling':ti,ab,kw OR 'right ventricle pulmonary artery coupling':ti,ab,kw OR 'rv-pa coupling':ti,ab,kw OR 'rvpa coupling':ti,ab,kw	721210
#5	'tricuspid annular plane systolic excursion'/de AND 'pulmonary artery systolic pressure'/de	427
#4	'heart ejection fraction'/exp OR 'heart stroke volume'/de OR 'heart work'/exp	272740
#3	#1 OR #2	478117
#2	echocardiogr*:ti,ab,kw OR (((heart OR cardiac) NEAR/2 (echogra* OR ultrasound* OR 'ultra sound*' OR ultrasonogra* OR 'ultra sonogra*' OR sonogra* OR ultrasonic OR 'ultra sonic')):ti,ab,kw)	321007
#1	'echocardiography'/exp	424539
